# Sessions of acupuncture and nutritional therapy evaluation for atrial fibrillation (Santé-AF): a randomised feasibility study

**DOI:** 10.1186/s40814-025-01604-w

**Published:** 2025-02-25

**Authors:** Karen Charlesworth, David J. Torgerson, Judith M. Watson

**Affiliations:** 1 Department of Health Sciences, University of York, Heslington, York YO10 5DD UK; 2 Northern College of Acupuncture, Micklegate, York YO1 6LJ UK

**Keywords:** Feasibility, Atrial fibrillation, Acupuncture, Nutrition, Nutritional Therapy, Complementary therapies, Trial, RCT, Pragmatic

## Abstract

**Background:**

Atrial fibrillation is a common cardiac arrhythmia, associated with debilitating symptoms and a decrease in health-related quality of life. Current treatments for atrial fibrillation may not provide symptomatic relief and are associated with risks and adverse responses. Large-scale trials are justified to investigate whether complementary therapies may improve symptoms and/or health-related quality of life in atrial fibrillation. To reduce the uncertainty of a future trial, a feasibility study was carried out.

**Design and methods:**

A three-arm, parallel-group, pragmatic randomised controlled feasibility study recruited 30 participants with paroxysmal AF aged 45–70 from NHS primary care, randomising to Group A (acupuncture + usual care), Group B (nutritional therapy + usual care) or Group C (usual care alone) using a 2:2:1 allocation ratio in favour of the interventions. Interventions were delivered by private practitioners. Seven feasibility objectives were investigated, including participants’ willingness to take part, appropriateness of eligibility criteria, participant retention and acceptability of interventions and study assessments. Additional exploratory feasibility objectives were investigated, including the effect of the COVID-19 pandemic and the safety of interventions. Data was analysed using descriptive statistics and reflexive thematic analysis, and the study used a sequential convergent mixed methods design to understand whether, and why, objectives were feasible and to make recommendations for a future trial.

**Results:**

Five feasibility objectives’ progression criteria were met, one did not meet its progression threshold and one was abandoned as infeasible to analyse but did not affect feasibility. Recommendations for a future trial include changes in eligibility criteria to reflect real-world populations and changes to assessment methods to reduce participant burden. Uncertainty remains around the effect on the feasibility of reversion to pre-COVID therapy and study assessment delivery, including a recommended longer follow-up.

**Conclusion:**

A future large-scale trial was found to be feasible with adjustments, but some uncertainty remains.

**Trial registration:**

ISRCTN13671984. Registered on June 04, 2020.

**Supplementary Information:**

The online version contains supplementary material available at 10.1186/s40814-025-01604-w.

## Key messages regarding feasibility

Uncertainties regarding feasibility were participants’ willingness to take part, the appropriateness of eligibility criteria, participant retention, acceptability of interventions and assessments, the utility of the CardioSTAT® ECG monitor and the overall acceptability of study participation. Other uncertainties were the effect of the COVID-19 pandemic on feasibility, and the safety of the interventions.

Key feasibility findings were that sufficient participants were willing to take part and were retained at follow-up, interventions were highly acceptable, study assessments were acceptable and participants’ overall experience of participation was highly acceptable. However, eligibility criteria were found to be inappropriate for a pragmatic trial, and the ECG monitor’s utility in a future trial was not analysable as planned, although this did not affect overall feasibility. The COVID-19 pandemic did not appear to affect overall feasibility, and participants did not experience any significant adverse reactions.

Feasibility findings have several implications for the design of a future trial chiefly that eligibility criteria should be widened to increase both trial recruitment and the generalisability of its results to the AF population, and study assessments should be held in person to minimise participant burden and measurement error. The remaining uncertainty around the feasibility of a future trial includes the effect of reversion to non-COVID-19 practice in both interventions and in study assessments and the effect of introducing one or more longer follow-ups to detect the effects of interventions over time.

## Background

Atrial fibrillation (AF) is a common cardiac arrhythmia with debilitating symptoms including breathlessness, palpitations and tachycardia, dizziness, fatigue and depression [1]. While up to 40% of the AF population is asymptomatic, the condition is associated with a sharp decrease in health-related quality of life (HRQoL) [2]. The burden on healthcare systems is substantial: in the UK, it is estimated that AF will account for up to 4.27% of total National Health Service (NHS) expenditure by 2040 [3]. AF is strongly associated with increased multimorbidity; a typical AF patient has an average of five comorbid conditions [4], thought to result from pathophysiological mechanisms shared between AF and conditions such as chronic obstructive pulmonary disease (COPD), heart failure and diabetes. Such comorbidities collectively contribute to remodelling of the cardiac substrate and worsen underlying cardiomyopathy, which in turn exacerbates both AF symptoms and the risk of stroke [5], also influencing responses to treatment [6]. Treatments, including medications and procedures such as electrical cardioversion and ablation, are not always successful in achieving normal sinus rhythm, and a high proportion of patients revert to AF within a year of treatment [7]. Refractory AF is treated with titrated or additive medications and/or multiple procedure cycles, which may show diminishing returns and an increase in treatment-related risk [1].

The last decade has seen an increase in research into complementary therapies for AF. Complementary therapies, operating on a holistic principle of “treating the patient, not the disease” [8], may be able to address a broad range of therapeutic targets including the comorbidities of the typical AF patient; this approach is congruent with the “look beyond the ECG, treat the patient” approach advocated by trials of conventional medicine focused on quality of life in AF [9]. Complementary therapies may also promote self-efficacy and a positive change in unhelpful lifestyle behaviours that contribute to AF severity [10]. A small number of trials have investigated various complementary therapies for symptoms and HRQoL in atrial fibrillation, covering yoga, Chinese herbal medicine, acupuncture/acupressure, osteopathy and nutrition/supplementation. In general, these studies are of low quality, with small sample sizes and moderate or high risk of bias. Additionally, most use a standardised protocol or a single isolated element of the therapy in question, limiting the studies’ generalisability by making results unrepresentative of real-world practice in which, typically, a number of therapeutic elements are combined to form individualised treatment plans responding to the patient’s specific needs. High-quality large-scale trials that take a pragmatic approach to investigate the effects of the real-world practice are therefore justified to investigate the effect of complementary therapies on symptoms and HRQoL in atrial fibrillation. To decrease some uncertainties associated with a future large-scale pragmatic trial of these two therapies in an AF population, a feasibility study was conducted. Two complementary therapies—acupuncture and nutritional therapy—were selected for investigation in a UK-based study on the grounds that both therapies are widely available to a high standard of safety and professional conduct [11–14].

## Design and methods

The Santé-AF feasibility study was a three-arm open-label parallel-group mixed-methods pragmatic feasibility randomised controlled trial. No interim analyses were carried out. The trial design was modified to mitigate the risk of COVID-19 infection, primarily by requiring nutritional therapists to work entirely online and by holding study assessments online. Cessation guidelines for the acupuncture arm were defined around the level of COVID-19 in the local area but were not activated as infection rates did not reach the thresholds. No important changes were made to methods after the start of the trial. The principal investigator (KC) was a qualified acupuncturist, although not delivering any trial treatments.

### Aim and feasibility objectives

The feasibility study aimed to reduce the uncertainty associated with several aspects of a future large-scale trial’s design (Table 1). Additional aspects of feasibility were explored, including the effect of COVID-19 on feasibility and the safety of interventions.

**Table 1:** Santé-AF feasibility objectives and thresholds (progression criteria)

Objective		Measurement	Feasibility threshold (progression criterion)	Data collection point	*n* included in analysis
Objective 1: Participants’ willingness to take part		Percentage of eligible participants randomised	≥90%	Baseline	29
Objective 2: Appropriateness of eligibility criteria		Percentage of identified participants eligible	≥60%	Pre-consent	220
Objective 3: Participant retention		Percentage of participants retained at follow-up	≥80%	Follow-up	25
Objective 4: Intervention acceptability		a. Participant satisfaction with group allocation @ 7–10 on 10-point scale	≥75%	Post-baseline	29
		b. Participant report of intervention acceptability @ 1–3 on 7-point scale	≥75%	Follow-up	20
Objective 5: Acceptability of study assessments		a. Completeness of main outcomes data	≥80%	Baseline; follow-up	30
		b. Participant report of intervention acceptability @ 1–3 on 7-point scale	≥90%	Follow-up	25
Objective 6: Utility of CardioSTAT® monitor		a. Participant report of monitor influence on participation @ 1–2 on 5-point scale	≥50%	Baseline; follow-up	(abandoned as unviable)
		b. Difference in number of AF episodes recorded by monitor and symptom diary	≤1 episode difference	Follow-up	(abandoned as unviable)
Objective 7: Experience of study participation		Participant report of overall study acceptability @ 1–2 on 5-point scale	≥75%	Follow-up	25

Quantitative feasibility thresholds were used as progression criteria and were primarily defined according to the precedents set by a survey of recent, similar pilot trials or feasibility studies. To set progression criteria, a short survey was conducted of recent trials or pilot/feasibility studies of broadly comparable interventions for cardiac conditions and/or AF comorbidities (see Additional file 2). Not all feasibility objectives were found in comparable studies; for instance, overall acceptability of study participation and levels of allocation satisfaction were not reported in any of the surveyed trials. Criteria followed recent guidance in being considered guidelines rather than absolute rules and being co-informed by qualitative analysis [15, 16].

### Sample size

A sample size was calculated for the feasibility study using Cocks and Torgerson’s 80% one-sided confidence interval approach; this bases the feasibility study sample size on the sample size of a future trial, giving a statistical basis for the decision to progress to a future trial [17]. First, the sample size of a future trial was calculated with a power of 80% and a significance level of 5% on its primary outcome measure, the Atrial Fibrillation Effect on Quality of Life (AFEQT) scale [18], using a target effect size of 9.8 points on the AFEQT scale and a corresponding standard deviation of 20.0 [18]. This resulted in a sample size for the future trial of ≈198 participants. Applying Cocks and Torgerson’s method gave a feasibility study sample size of ≈24 participants across three groups or 30 participants when adjusted for 20% attrition and rounded for convenience.

### Interventions and study setting

**Fig. 1:**
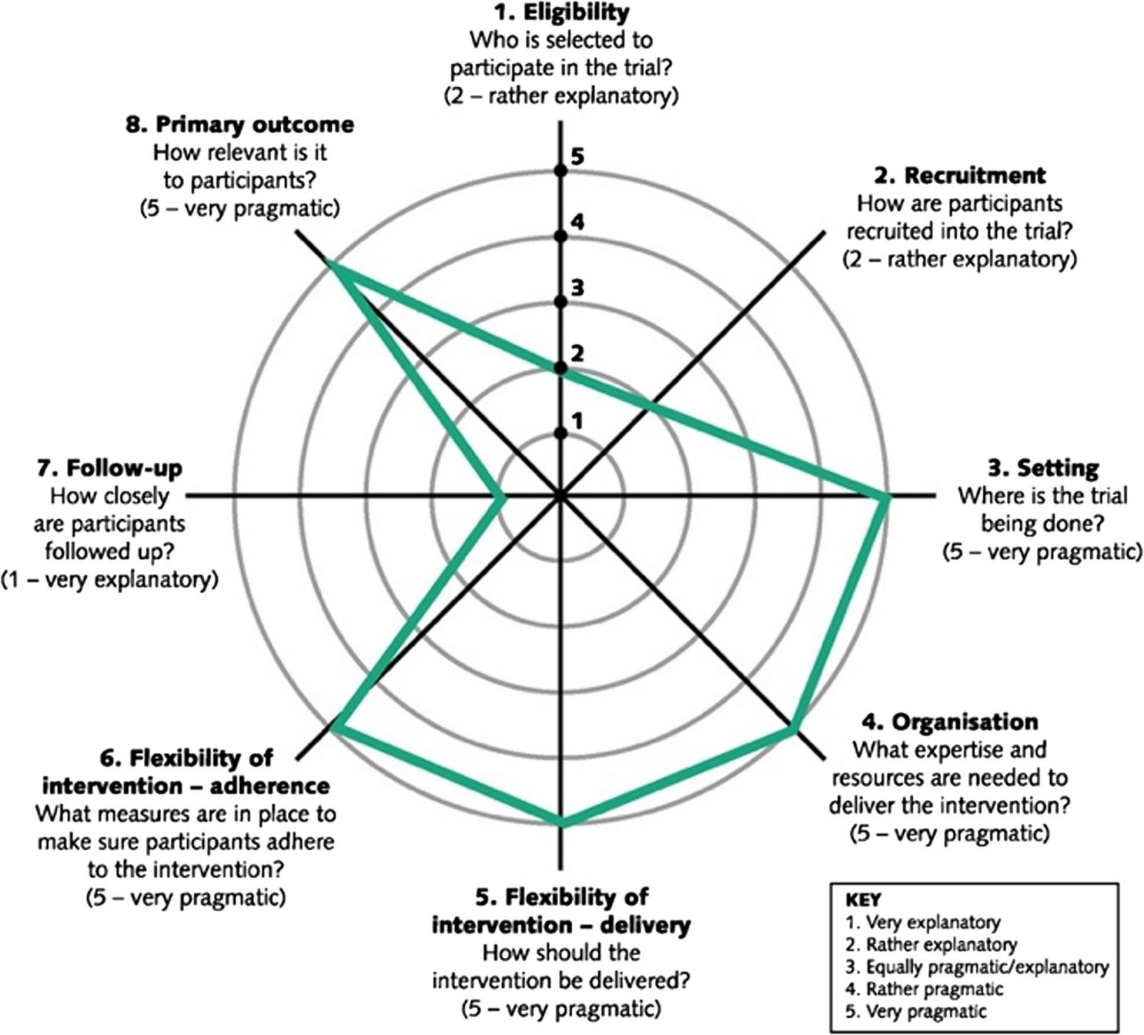
PRECIS-2 “pragmascope” for a future trial

As practised in the UK, both acupuncture and nutritional therapy (NT) rely on an individualised approach in which the patient is seen as a unique, complex whole shaped by their own history, beliefs and wider social, geopolitical, educational, cultural and financial systems; treatments are formulated to respond to this unique, complex whole patient and to adapt dynamically to their changing needs, often moment by moment during a treatment process [13, 14]. To investigate the effectiveness of individualised therapies as practised in routine settings, a large-scale trial should take a pragmatic approach to design [18], particularly in the dimension of intervention delivery (Fig. 1). Santé-AF therefore required practitioners of both therapies to follow their usual scope of practice and routine processes. Practitioners kept log books recording details of treatment approaches and components of the intervention used for each participant. Analysis of this data is reported in a separate paper but follows the scope of practice set out by the British Acupuncture Council and the British Association of Nutrition and Lifestyle Medicine [13, 18]. For an overview of treatment strategies, see the “Exploratory analysis: Treatment strategies” section.

Acupuncture (Group A) is thought to have originated in China between 100 and 200 BCE [18] and involves the insertion of filiform needles at “acupoints” on the body. Western interpretations of acupuncture’s mechanisms of action in an AF context include stimulation of the vagus nerve via its auricular branch, which reduces cardiac sympathetic activity and promotes parasympathetic activity, to which extent it is thought to have a cardioprotective function and may reverse atrial remodelling and attenuate AF symptoms [18]. In Santé-AF, acupuncture was defined as the Traditional Chinese Medicine (TCM) style of acupuncture and various submodalities (including cupping, electroacupuncture, moxibustion and lifestyle advice) as recognised and insured by the British Acupuncture Council (BAcC). Participants attended practitioners’ usual private clinics, and practitioners aimed for a regime of once-weekly treatments of 45–60 min over 10 weeks, although this was varied to meet the needs of patients as per routine practice. An initial discussion with practitioners was held to determine the average duration of a course of treatment in regular practice, which was agreed to be 10 weeks for eight treatments; this was different from the 12-week window allowed for nutritional therapy consultations, but the difference was justified as reflecting routine practice in both therapies. Treatments were delivered according to the BAcC’s COVID-secure guideline current during the intervention period, including enhanced cleaning, ventilation, use of personal protective equipment and enhanced hand-washing [18].

Nutritional therapy (NT) (Group B) is a new and developing complementary medicine, also known as “personalised nutrition”. Based on consultation and functional/genomic testing, patients receive individualised plans including dietary advice, eating behavioural advice, supplementation and lifestyle advice; practitioners adjust plans as required and support patients to follow them [18]. For atrial fibrillation, mechanisms of action include “nodal points” affecting AF pathogenetic processes, such as inflammation, oxidative stress, autoimmune mechanisms and visceral adiposity [18]. Recent literature hypothesises that metabolites derived from gut microbiota may regulate cardiac substrate and so may be leveraged in a therapeutic strategy [18]. In Santé-AF, NT was defined as corresponding with the core curriculum for NT as set out by the UK Complementary and Natural Healthcare Council (CNHC) [18] and modified by the study’s budget, which precluded the use of functional/genomic testing or supplementation, meaning that the nutritional therapists (NTs) in the study focused on dietary strategies and lifestyle advice. To mitigate the risk of COVID-19 transmission, consultations were delivered online using practitioners’ usual video-conferencing software, and practitioners aimed for a regime of three consultations of 60–90 min over 12 weeks, although appointment durations and intervals varied to meet the needs of patients as per routine practice. An initial discussion with practitioners was held to determine the average duration of a course of treatment in regular practice, which was agreed to be 12 weeks; this was different from the 10-week window allowed for acupuncture treatments but the difference was justified as reflecting routine practice in both therapies.

Usual care (UC) (Group C) for Santé-AF comprised the care pathway as defined by the National Institute for Health and Care Excellence (NICE) guideline on AF (NG196) [18], delivered via primary care practices in conjunction with hospital consultants and/or other healthcare professionals.

#### Participant eligibility criteria, informed consent and recruitment

Participants were eligible to take part if they were aged ≥ 45 and ≤ 70, had a diagnosis of AF dated 6–60 months previous to recruitment and had self-detectable paroxysmal atrial fibrillation of at least weekly frequency. To attend study assessments and nutritional therapy consultations, participants had to have home broadband and a device capable of video conferencing. Participants were excluded if they had valvular AF, any type of active implantable device including a pacemaker, diagnoses of kidney disease levels 4/5, terminal or severe illness, blood-clotting disorders, eating disorders or COVID-19 vulnerability. Full eligibility criteria, with justifications, can be found in Supplementary File 1.

Four primary care practices (participant identification centres or PICs) in the Vale of York, UK, searched their records to identify patients. Potentially eligible patients were contacted by post by the PIC to ascertain their willingness to receive more information about the study. Willing patients received a participant information sheet and consent form and were contacted by the principal investigator (KC) to answer any queries. Following this, patients who were willing to participate returned a signed consent form and underwent a second stage of screening to check eligibility criteria not available via medical records.

#### Randomisation

Eligible participants were randomised to one of three groups (A, acupuncture + UC; B, nutritional therapy + UC; C, UC alone) using a 2:2:1 ratio in favour of the intervention groups. This allocation ratio was used to increase power in the two intervention groups to maximise the ability to detect differences in effect size at follow-up and also to allow for a more robust investigation of specific covariates within and between groups. Randomisation was carried out by the principal investigator (KC) using the Sealed Envelope Simple + online facility [18], incorporating a block design with a list length of 30 and randomly permuted block sizes of 5 and 10. Allocation was concealed from the investigator until the point of assignment. A subgroup of 10 participants, randomised using the RAND function in a Microsoft Excel spreadsheet with the same allocation ratio as the primary groups, was allocated to wear a two-lead ambulatory ECG monitor [18] for 7 days following the baseline and follow-up assessments. A further subgroup of 16 participants was selected for semi-structured interviews at baseline and follow-up, based on a maximum variation sampling strategy.

#### Blinding

Pragmatic trials largely do not incorporate blinding of participants, and the nature of the interventions themselves did not allow for the blinding of participants or practitioners. The principal investigator carried out enrolment, randomisation and most of the assessment and was aware of group allocation throughout. Icentia Limited, supplier of the CardioSTAT® ECG monitor, conducted monitor data extraction without knowledge of the intervention received.

#### Data collection

Two study assessments were carried out, once at baseline and once at an end-of-treatment follow-up approximately 3 months after the start of treatment. All assessments were held online to minimise the risk of COVID-19 transmission. Assessments tested the feasibility of collecting data required by a future trial and collected additional data to directly answer other feasibility objectives. There were no changes to trial assessments or measurements after trial commencement.

All participants were asked to complete a questionnaire covering demographics, COVID-19, AF symptoms and quality of life, general health, healthcare resources usage and self-care/lifestyle, along with reasons for participation, experience of participation, experience of assessment and experience of therapy received. Two validated measures, the EQ-5D-5L and the AFEQT scale, were incorporated into the questionnaires [18, 18]. Participants were also asked to provide anthropometric data including height, weight, blood pressure and waist/hip measurements using calibrated equipment provided, along with details of current medications. Semi-structured interviews were held with a subgroup (*n* = 16), covering topics including diagnosis, treatment and the experience of living with AF, and questions related to study participation, including study/assessment experience and the effect of the COVID-19 pandemic on participation acceptability. Participants randomised to wear the ECG monitor (*n* = 10) were guided to self-fit the monitor during assessments.

### Data analysis

Quantitative data analysis focused on progression criteria, using descriptive statistics including means and standard deviations, and count data including percentages, to ascertain whether a given domain met its progression threshold. Qualitative data was analysed using reflexive thematic analysis [18], to supplement the quantitative data and inform recommendations for a future trial. Both types of data were synthesised using joint display tables [18] in relation to their objective.

## Results

### Participant flow

**Fig. 2:**
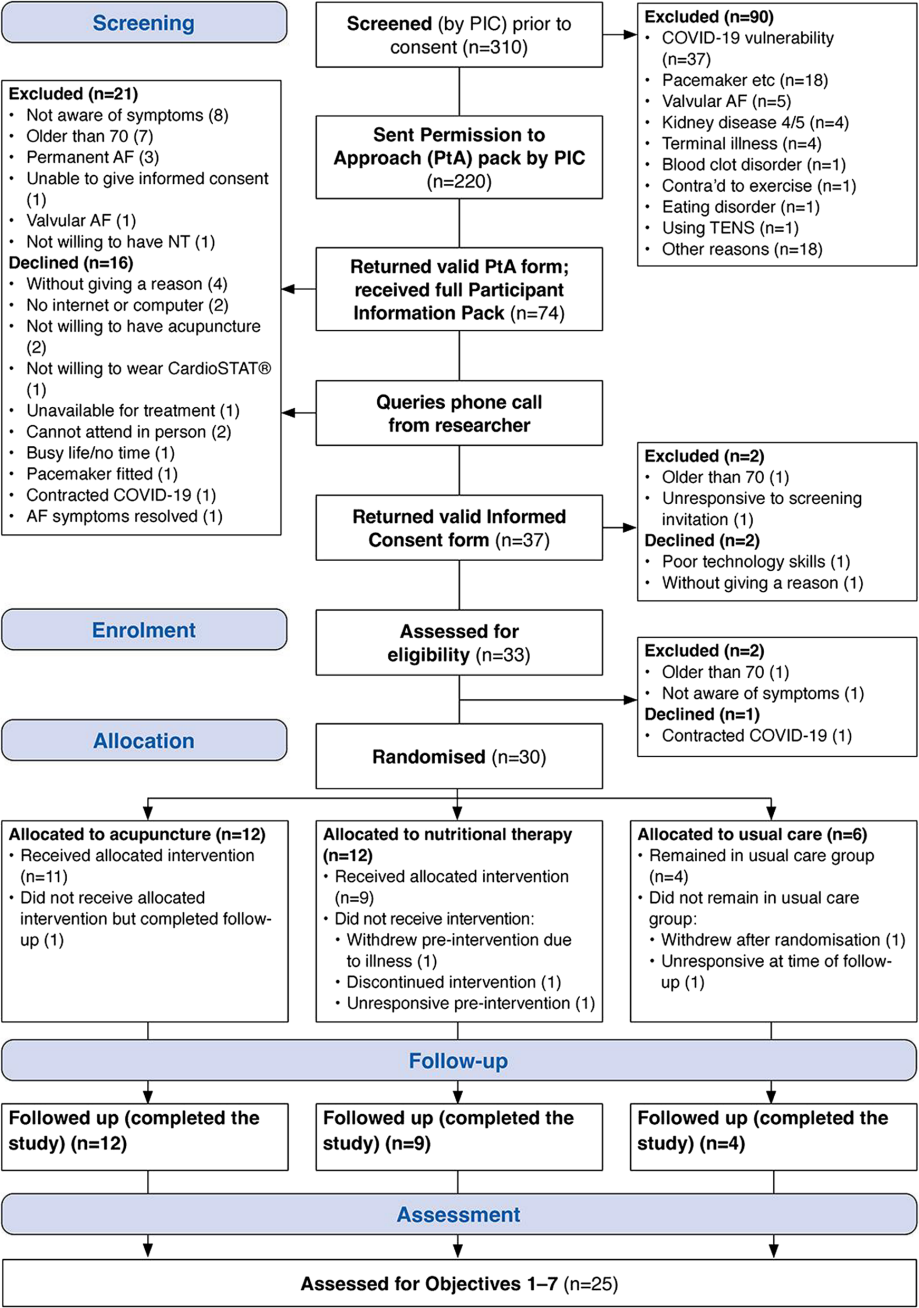
Participant flow

The study began recruitment in September 2021. Randomisation took place between November 12, 2021, and February 01, 2022, and follow-up assessments were carried out between February 22, 2022, and May 19, 2022. Participant flow is shown in Fig. 2.

### Participant baseline characteristics

Participants’ characteristics at baseline are shown in Table 2.

**Table 2:** Participants’ baseline characteristics

	Acupuncture (*n* = 12)	Nutritional therapy (*n* = 11)	Usual care (*n* = 6)
Age	61.1 ± 4.9	61.0 ± 5.5	56.3 ± 8.9
Sex			
Male	7 (24.1%)	10 (34.5%)	5 (17.2%)
Female	5 (17.2%)	1 (3.4%)	1 (3.4%)
Ethnicity			
White	12 (41.4%)	11 (37.9%)	6 (20.7%)
Average duration of AF, months	41.3 ± 23.9	38.8 ± 17.5	28.3 ± 18.8
Average AFEQT score (higher = better)	71.1 ± 20.4	76.2 ± 13.9	63.4 ± 32.3
BMI	31.3 ± 6.1	28.4 ± 5.1	31.8 ± 3.7
Waist-hip ratio			
Male	0.97 ± 0.03	1.00 ± 0.06	1.02 ± 0.04
Female	0.89 ± 0.04	0.92*	0.90*
Blood pressure, mmHg			
Systolic	142.8 ± 23.4	146.4 ± 20.8	144.0 ± 18.5
Diastolic	92.8 ± 6.9	94.9 ± 12.4	91.3 ± 8.3
Units alcohol/week	23.9 ± 24.9	22.6 ± 17.1	14.0 ± 16.4
Average number comorbidities per participant	1.1 ± 1.4	1.3 ± 1.4	1.8 ± 2.3
Average number of medications per participant	3.8 ± 2.3	3.7 ± 2.0	3.8 ± 1.9

Figures are means ± standard deviation or count data (followed by the percentage of total *N* in the trial)

*Figure applies to the single female participant in this group (therefore, figure is not a mean, and no standard deviation is given)

### Results by objective

#### Overall feasibility

Table 3 shows the summary judgement on feasibility based on quantitative progression thresholds. Of the seven progression criteria, five were met or bettered, one did not meet its progression threshold and one was abandoned as the analyses were unviable.

**Table 3:** Overall feasibility

Objective	Measurement	Progression threshold	Level achieved	Feasibility of domain
**Objective 1:** Participants’ willingness to take part	Percentage of eligible participants randomised	≥ 90%	96.8%	Yes
**Objective 2:** Appropriateness of eligibility criteria	Percentage of identified participants eligible	≥ 60%	14.1%	No
**Objective 3:** Participant retention	Percentage of participants retained at follow-up	≥ 80%	83.3%	Yes
**Objective 4:** Acceptability of interventions	a. Participant satisfaction with group allocation @ 7–10 on a 10-point scale	≥ 75%	75.0%	Yes
	b. Participant report of intervention acceptability @ 1–3 on a 7-point scale	≥ 75%	95.0%	Yes
**Objective 5:** Acceptability of assessments	a. Completeness of main outcomes data	≥ 80%	90.0%	Yes
	b. Participant report of assessment acceptability @ 1–3 on a 7-point scale	≥ 90%	92.0%	Yes
**Objective 6:** Utility of CardioSTAT® monitor	a. Participant report of monitor influence on participation @ 1–2 on a 5-point scale*	≥ 50%	–	[Analysis unviable]
	b. Difference in the number of AF episodes each recorded by monitor and symptom diary	≤ 1 episode difference	–	[Analysis unviable]
**Objective 7:** Participant experience of study participation	Participant report of overall participation acceptability	≥ 75%	96.0%	Yes

### Objective 1: Participants’ willingness to take part

The total number of participants who were eligible after the screening was 31, of which 30 were randomised (96.8% of the eligible total). The progression threshold for this domain was ≥ 90%; this objective was judged feasible for a large-scale trial without the need for changes. No patient declined to participate due to a perception of COVID-19 risk, although two declined after contracting the virus. Three participants declined to participate due to not having a computer or sufficient computer skills, or adequate Internet capacity.

Twenty-nine participants were asked about their reasons for participating. Participants’ most important reasons were curiosity about acupuncture, and wanting to help others with AF (each *n* = 6, 20.7%):

It’s not just for me … there’s lots of people have this thing (2/163/A)

Mainly to develop better cures for the problem (3/978/A)

Sixteen patients gave reasons for declining to participate, of which the most frequent were not having a computer, Internet or technology skills (*n* = 3), or not willing to have acupuncture (*n* = 2).

### Objective 2: Appropriateness of eligibility criteria

“Appropriateness” was defined as the ability of the criteria to produce a sufficient cohort of patients to meet the required sample size (*n* = 30). Thirty-one (14.1%) of the 220 PIC-identified eligible patients were eligible at the second screening stage, meaning the domain did not meet its progression threshold.

COVID-19 vulnerability excluded 37 of 2744 patients (1.4%) at the PIC search/screen stage, and a further three were excluded at the second screening stage due to not being able to use online technology for assessments (0.1%).

### Objective 3: Participant retention

Twenty-five of 30 (83.3%) randomised participants were retained at follow-up. This objective was judged feasible without needing amendment.

Five participants withdrew. One (Group B) withdrew after randomisation but before baseline assessment due to a terminal diagnosis. One (Group B) became uncontactable after baseline assessment. One (Group B) discontinued the intervention and was uncontactable. One (Group C) did not complete the follow-up due to repeated cancellations of the appointment, eventually becoming uncontactable. One withdrew due to being randomised to Group C. No participant withdrew due to COVID-19.

Participants’ most prevalent reasons for remaining in the study included wanting to help others with AF (*n* = 12; 48.0%), therapies’ effectiveness (*n* = 6; 30.0%) and 13 participants (all from the active intervention groups) regarding themselves as, on principle, “not a quitter… if you stand up to do something, you do it” (1/661/A) and “seeing [things] out” (1/557/A).

### Objective 4: Acceptability of interventions

For objective 4a, 28 participants (96.5%) responded to a post-randomisation SMS poll requesting their level of satisfaction with group allocation on a scale of 1–10 (7–10 = high satisfaction). Twenty-one (75.0%) reported a high level of satisfaction with their group allocation (acupuncture = 11, NT = 7, UC = 3). This objective met the progression threshold without the need for amendment.

For objective 4b, the 20 participants remaining in the intervention groups at follow-up were asked to rate their intervention’s acceptability on a scale of 1–7 (1–3 = high acceptability). Nineteen (95.0%) participants reported high acceptability (acupuncture = 11, NT = 8) which was explicitly related to positive perceived effects on AF symptoms including exercise tolerance (2 acupuncture, 1 NT), dyspnoea (3 acupuncture, 2 NT) and palpitations (4 acupuncture, 2 NT). Perceived positive effects on other symptoms included musculoskeletal problems (3 acupuncture, 1 NT), (positive) weight loss (3 NT), “mental attitude” (2 acupuncture), peripheral oedema (1 acupuncture), blood pressure (1 acupuncture), irritable bowel syndrome (1 NT); bowel irregularity (1 NT) and Parkinson’s tremor (1 acupuncture). Interventions were also noted as having positive effects on general health and wellbeing (5 acupuncture, 2 NT), sleep (4 acupuncture, 1 NT) and energy (5 acupuncture, 2 NT). This objective met the progression threshold without the need for amendment.

Less acceptable aspects of the interventions included booking and making time for appointments: this was more pronounced in the acupuncture participants, who had to accommodate a series of eight appointments and associated travelling time (travel expenses were subsidised), compared with three appointments and no travelling time for the Nutrition group:

It was a little bit awkward at times because I had to cancel stuff and move stuff … so there was a bit of pressure there (2/761/A)

I got there dead on time one day… when you get back of a tractor going at 20 mile an hour… our roads aren’t the best for passing on (3/156/A)

COVID-19 made little difference to intervention acceptability. Acupuncture group participants were reassured by acupuncturists’ compliance with the BAcC COVID-secure guidelines, while NT group participants “would prefer face-to-face” appointments (2/481/NT) but appreciated the convenience of being online and, in general, did not find the technology difficult to use.

### Objective 5: Acceptability of study assessments

For objective 5a, completeness of data returned for the main outcomes (EQ-5D-5L; AFEQT; CardioSTAT® 7-day ECG monitor; 7-day symptom diary) was analysed as a proxy for assessment acceptability. Overall completeness of aggregated data (including data missing due to participant attrition) for all four outcomes at baseline and follow-up was 90.0% ± 7.2%, comparing favourably with similar studies [18, 18]. This objective was feasible without the need for amendment.

For objective 5b, the 25 participants remaining in the study at follow-up were asked to indicate the overall acceptability of both assessments on a scale of 1–7 (lower numbers = greater acceptability), with 24 (92.0%) rating acceptability at 1–3 on the scale; this objective was deemed feasible without adjustment.

The need to hold assessments online due to COVID-19 impacted assessments’ acceptability. Qualitative data for this objective revealed that participants disliked taking their own anthropometric measurements while online: “tricky” (2/515/UC); “difficult when… having headphones on & wearing a dressing gown” (1/557/A). Nine participants took measurements out of camera view, giving a variety of reasons such as room temperature, privacy and dignity; this made it difficult to verify that the correct procedure had been followed, giving the potential for measurement error.

### Objective 6: Utility of CardioSTAT® monitor

“Utility” for this objective was defined as the monitor’s role in the feasibility of a future trial. For objective 6a, the nine CardioSTAT® wearers remaining at follow-up were asked to rate the influence of the monitor on their decision to participate, on a scale of 1–5 (1 = strongest influence); one participant (11.1%) gave a rating of 1, while the remaining eight gave a rating of 3 (neither influential nor non-influential). The progression criterion for objective 6a was ≥ 50% of participants finding the monitor highly influential in their decision to participate, meaning that this progression threshold was not met; however, it was recognised that this objective was more exploratory than deterministic, and the progression threshold was not applied.

For objective 6b, the agreement between the monitor and participants’ symptom diaries, expressed as the number of AF episodes recorded by each method, was tabulated; however, a high proportion of non-analysable data from the CardioSTAT® (periods of random length in which levels of noise obscured the electrodes’ signal) made this analysis unworkable, and the progression threshold could not be applied.

The COVID-19 pandemic appeared not to influence the experience of the CardioSTAT® for participants, although participants’ self-fitting of monitors during online assessments may have been partly responsible for the high proportion of noise that rendered comparison with the symptom diaries infeasible.

Exploratory analysis of the data for this objective revealed other findings of interest for a future trial (Table 4). All 10 participants in the analysis had recorded symptoms where AF was not present and/or had not recorded symptoms where AF was present, suggesting that these participants could not reliably detect their AF. Additionally, some participants who were coded on the medical record as having paroxysmal (episodic) AF in fact experienced 100% AF during analysable data periods; although the analysable data period was less than seven days (the threshold at which AF is judged to progress from paroxysmal to persistent), this is nonetheless suggestive of undetected progression.

**Table 4:** Active AF and perceived symptoms per participant

	CardioSTAT® data		Symptom diary data		
	Active AF recorded (minutes)**	AF % of analysable data	Perceived symptoms recorded (minutes)	# entries recorded	# entries corresponding to active AF**
1/661/A baseline*	8197.6	100.0	572.7	10	8
1/661/A follow-up	6475.1	100.0	690.0	5	2
1/884/UC baseline*	0.0	0.0	114.6	3	0
1/884/UC follow-up	9937.0	100.0	5.0	6	6
2/174/NT baseline	0.0	0.0	75.0	5	0
2/256/A baseline	1720.2	100.0	5.0	1	0
2/256/A follow-up	1181.7	100.0	0.0	0	0
2/481/NT baseline	4705.5	100.0	90.0	4	1
2/481/NT follow-up	8399.6	100.0	35.0	2	2
2/515/UC baseline	285.0	2.8	150.0	3	2
2/515/UC follow-up*	112.6	1.1	305.5	8	2
2/761/A baseline	10,287.1	100.0	0.0	0	0
2/761/A follow-up	9800.4	100.0	480.0	2	2
2/964/NT baseline	661.4	6.5	49.6	7	2
2/964/NT follow-up	235.5	2.3	41.0	6	2
4/385/NT baseline	0.0	0.0	165.0	4	0
4/385/NT follow-up	0.0	0.0	0.2	1	0
4/910/A baseline	9734.6	100.0	125.0	5	5
4/910/A follow-up	10,168.3	100.0	10.0	1	0

*Some or all durations missing from the symptom diary. Mean of all other observations used to impute missing values

**Derived from analysable data only

### Objective 7: Experience of study participation

Participants retained at follow-up were asked to rate the overall acceptability of study participation on a scale of 1–7 (lower numbers = greater acceptability), with 24 of 25 (96%) participants giving a rating of 1–3. All participants gave short, generally positive views of their experience: “very interesting and helpful” (2/163/A), “very informative experience” (3/806/NT) and “enjoyed taking part” (4/693/NT). This objective was feasible without the need for amendment. COVID-19 did not affect the overall acceptability of participation.

### Exploratory analysis: Safety of the interventions

Participants in the intervention groups were asked to report on any undesired side-effects of treatment, and practitioners were asked to note serious adverse events, adverse reactions or unconnected adverse events unconnected with the study treatment. No serious adverse events or unconnected adverse events were reported by participants or practitioners; nine adverse reactions were reported by both, of which eight were related to the Acupuncture group and were minor, transient and self-limiting (being more tired than usual (*n* = 3), bruising (*n* = 3), nerve sensation (*n* = 1), slight headache (*n* = 1)). Only one patient in the NT group reported an adverse reaction, relating to muscle fatigue and tiredness worsening over 4 weeks; the participant’s dietary advice was adjusted (part of routine practice) to maintain the beneficial effects of treatment already experienced while alleviating the adverse reaction. The participant chose to continue in the study.

### Exploratory analysis: Within-group means

Group mean scores for a future trial’s primary outcome measure (the AFEQT questionnaire) were calculated at baseline and follow-up (Table 5). The normality of data distribution was assessed visually with a Wilcoxon signed-rank test histogram and found to be non-normal in the usual care group, possibly induced by the small group size.

**Table 5:** AFEQT questionnaire: group means at baseline and follow-up

	Acupuncture (*n* = 12)	Nutritional therapy (*n* = 9)	Usual care (*n* = 4)
Baseline	71.1 ± 20.4	76.2 ± 13.9	63.4 ± 32.3
Follow-up	81.3 ± 13.6	85.2 ± 12.5	76.6 ± 20.3
Figures are mean ± standard deviation. Follow-up duration averages 94 days. Higher scores = better			

### Exploratory analysis: Treatment strategies

Santé-AF was highly pragmatic in the dimension of intervention delivery, and practitioners followed a scope of practice characteristic of routine delivery. Log books were analysed to indicate the most frequent treatment strategies, and the results are summarised here.

For the 10 participants followed up in the nutritional therapy group, the most frequent nutritional strategy was to balance blood sugar levels (*n* = 10), followed by components of the Mediterranean diet (*n* = 7) and a low-carbohydrate diet (*n* = 5). Specific frequent recommendations included an increase in seeds, nuts and small wild oily fish; a reduction in refined and concentrated sugars; a general reduction in pro-inflammatory foods; an increase in vegetable and plant-based foods; and a reduction in processed foods and alcohol consumption. The mean number of treatments per participant was 2.8 of a maximum of 3 over 12 weeks’ duration. The mean length of consultation was 76 min at a mean interval of 33.4 days. A range of lifestyle advice was offered, including behavioural advice relating to diet (77.8%), exercise (63.0%), rest/sleep (63.0%), relaxation (66.7%) and working habits (11.1%).

For the 11 participants followed up in the acupuncture group, the most frequently-used points were Nèiguan P6 (7.40%), Shénmén HT7 (5.13%), Jiuwéi REN15 (4.44%), Shanzhong REN17 (4.21%), Jùquè REN14 (3.99%), Zusanli ST36 (3.76%), Táichong LIV3 (3.64%), Sanyinjiao SP6 (3.42%) and Xinshu BL15 (2.96%). The mean number of treatments per patient was 7.82 of a maximum of 8 over 10 weeks’ duration. The mean length of the appointment was 54 min, with a mean needle retention time of 21 min and a mean of 16 needle insertions. All needles were stainless steel, branded Seirin, Tewa, Acurea, Carbo, and Acuplus. Needle lengths varied from 13 to 40 mm, with gauges of 0.16–2.0, inserted 0.5–10 mm, all seeking De Qi from light to strong. Manual stimulation was used in all treatments except one which used electroacupuncture on four needles. Acupressure or massage was used in four treatments (4.55%) and an infrared heat lamp in 13 treatments (14.77%); no other adjunctive modalities were used. A range of lifestyle advice was offered, including diet (30.1%), exercise (21.6%) and rest/sleep (17.0%).

## Discussion

Where comparable measures were used, this study’s objectives’ feasibility and results were broadly comparable or improved upon those of similar feasibility studies, including recruitment (19–82% enrolled compared with 96.8% for the current study) [18, 18, 18, 18] and retention (74–78% retained at an average 12-week follow-up compared with 83.3% for the current study) [18, 18, 18, 18]. However, retention in this study compares unfavourably with median retention rates of 88% in large-scale trials published in NIHR journals [18]. Other dimensions of feasibility that were bettered against similar studies included the acceptability of both interventions [18] and the acceptability of study assessments [18, 18].

Participants’ baseline characteristics (Table 2) show notable imbalances between groups, including age, sex, average duration of AF, average AFEQT score, reported alcohol consumption and average number of comorbidities per patient. All these characteristics may influence response to treatment, with particularly strong associations between AF duration, age and number of comorbidities, and any treatment effect. It is recommended that a future trial should either stratify its randomisation to balance these characteristics or include them as a covariate in analyses if they appear at baseline.

The single unmet progression threshold (objective 2, appropriateness of eligibility criteria) did not significantly affect the validity of this study’s findings, since it represents one of a total of 10 thresholds and may be straightforwardly addressed by widening a future trial’s eligibility criteria, which would also have the effect of increasing the generalisability of findings to the AF population. Recommendations include extending the upper age limit to 85 years and removing the 60-month limit; this combination of measures would enfranchise an additional section of the population with an AF prevalence of 26.4% compared with the average 4.7% in the feasibility study’s ≥ 45 ≤ 70 age range [18]. Table 4 also suggests that the inclusion criterion of paroxysmal AF should be removed to avoid randomising participants whose AF has progressed without detection. Finally, unless a future trial was to take place under pandemic conditions, the eligibility criteria relating to COVID-19 should be discarded. However, widening eligibility criteria potentially introduces greater heterogeneity in the cohort, including heterogeneity of comorbidities that may influence response to treatment.

The random periods of non-analysable data generated by the CardioSTAT® did not allow the measurement of absolute levels of AF during the seven-day recording period. However, the use of objective measurement for AF is not common in trials of interventions for AF, with most trials relying on self-report as a measurement; as participants were also self-reporting symptoms in this study, the abandonment of analysis for objective 6 did not affect the feasibility of a future trial, nor the comparability of its results with other trials of interventions. However, the group means of active AF recorded by the CardioSTAT® may still be an informative analysis at follow-up in a future trial. Despite the device’s inability to measure the absolute extent of active AF, a future trial will assume that (i) sample size and randomisation have balanced between-participant differences at baseline (and therefore that within-participant differences are similarly balanced between groups at follow-up) and (ii) there are no significant differences in the average proportion of analysable CardioSTAT® data between groups (as these differences are random between participants in any case, and there is no other reason why a group-specific reason for differences should exist). To the extent that these assumptions are valid in a future trial, it is also valid to use the CardioSTAT® to compare between-groups differences at follow-up.

Notwithstanding the inability to carry out the analysis as planned, the comparison of monitor data with the symptom diary (objective 6b) did reveal findings of interest for a future trial: chiefly, it demonstrated that perceived symptoms cannot reliably be conflated with active AF. Although there may be additional reasons for participants not recording their perceived symptoms in the symptom diary (such as questionnaire fatigue, emotional responses to symptoms, and inconvenience of recording), this finding is congruent with recent research that found the experience of symptoms to be highly heterogeneous across the AF population, with an older population in particular noticing their AF less [18, 18]. This suggests that a self-report symptom diary may not be an accurate measure of active AF episodes across the AF population, particularly in the older population included in the adjusted age range recommended above. Nonetheless, the diary may have independent value in a future trial if positioned explicitly as a record of participants’ *perception* of symptoms, as distinct from the objective reading provided by the monitor. Perception of AF symptoms has been linked to poor HRQoL [18] and higher rates of emotional distress, including suicidal ideation, than in healthy populations [18, 18, 53]; a decrease in perceived symptoms may therefore indirectly benefit HRQoL [18]. It is therefore recommended that a future trial should retain the symptom diary, with a view to potentially conducting an analysis of the correlation between the level of perceived symptoms and HRQoL, as measured by either or both the AFEQT scale and the EQ-5D-5L.

Other recommendations to enhance the feasibility of a large-scale trial include the recruitment of practitioners closer to the participants’ homes to reduce travelling times and participant burden and the in-person conduct of study assessments led by a suitably trained healthcare professional to reduce participant burden and increase measurement validity.

All recommendations are shown in Table 6.

**Table 6:** Santé-AF recommendations for changes to the methods of a future trial

Objective		Recommendation for future trial	Rationale
Objective 1: Participants’ willingness to take part		No recommendations	n/a
Objective 2: Appropriateness of eligibility criteria		Either stratify randomisation on characteristics that may affect response to treatment or include as covariates in analyses	To prevent baseline imbalance between groups
		Widen eligibility criteria by extending the upper age limit to 85 years, removing 60-month limit on diagnosis length, removing the paroxysmal AF criterion and discarding eligibility criteria relating to COVID-19	To enfranchise a cohort more representative of the general population with AF
Objective 3: Participant retention		No recommendations	n/a
Objective 4: Intervention acceptability		Recruit practitioners closer to participants’ homes	Reducing travelling times and the general burden of compliance
Objective 5: Acceptability of study assessments		Retain both symptom diary and CardioSTAT® ECG monitor as measurements of symptomaticity	Ability to compare between self-report and objective measures
		Carry out study assessments in person at a study centre, with a suitably trained healthcare professional taking anthropometric measurements and blood pressure, and fitting CardioSTAT® ECG monitor	To reduce participant burden, increase acceptability of study assessments, and increase measurement validity
Objective 6: Utility of CardioSTAT® monitor		See Objective 5 above	–
Objective 7: Experience of study participation		No recommendations	–
General recommendations		Emphasise to participants the confidentiality of reporting; data collectors and outcomes assessors should be blinded to treatment allocation	To avoid reporting biases in a pragmatic trial assessed partly by self-report
		Include longer follow-ups than in this feasibility study	To demonstrate any effects of acupuncture and nutritional therapy over a longer term
		Design as a multi-centre trial	Enhance generalisability
		Include supplementation and functional testing in the scope of Nutritional therapy	Enhance real-world applicability of trial findings

### Effect of COVID-19

The COVID-19 pandemic introduced modifications to the study design, representing multiple limitations that impacted all feasibility objectives. Some feasibility objectives appeared only minimally affected by the pandemic, including the acceptability of intervention and participants’ general experience of participation. Other objectives more substantially affected included those related to recruitment, while the feasibility study did recruit its target number of participants, eligibility was impacted by adding extra eligibility criteria such as the requirement to exclude those classified as shielding or clinically vulnerable to COVID-19. The transition to online delivery of nutritional therapy and online study assessments also introduced inclusion criteria regarding home broadband of sufficient capacity to sustain video conferencing, and a level of technology literacy that may have excluded a number of study participants. It may be speculated that in a post-pandemic scenario, the removal of these eligibility criteria would positively affect recruitment, but this speculation cannot be supported by the data derived from the feasibility study.

The main effect of holding study assessments online due to COVID-19 was to introduce a potential for measurement error in the collection of anthropometric and ECG monitor data that would be obviated in a post-pandemic future trial: participants would be invited to attend a study centre where a qualified healthcare professional would fit the CardioSTAT® monitor and take weight, height and blood pressure measurements. Most participants expressing responses to online study assessments felt they would prefer data collection to be done in this manner; this would improve the acceptability of study assessments.

Arguably the most substantial area of uncertainty remaining for a future trial concerns the COVID-19-related changes in the mode of delivery for both therapies, as neither therapy was delivered in a manner representative of a future trial. Following the official end of the pandemic in April 2022, acupuncturists relaxed the BAcC COVID-secure code and returned to pre-pandemic levels of handwashing, patient segregation and use of PPE, while most nutritional therapists reverted to a modus operandi of some or all appointments conducted in person, meaning that a future trial would not necessarily use online methods of delivery. The effect of reversion to pre-COVID therapy delivery, and in-person study assessment conduct, remain substantial areas of uncertainty for a future trial.

Other feasibility studies for acupuncture related to other healthcare conditions have shown a standard delivery of the therapy to be feasible and acceptable [18, 18, 18, 53, 18]; however, there are no known feasibility studies or pilot trials of nutritional therapy, and such an assumption may therefore not be warranted. Additionally, there are no known studies testing the acceptability of trial assessments under COVID-19 conditions. With this in mind, it may be considered advisable to conduct a small, limited feasibility assessment as an internal pilot study, specifically targeted at those aspects of this study that were affected by COVID-19. This would allow future triallists to check the assumption that removing COVID-19-related barriers enhances the acceptability of both interventions and study assessments [53].

### Safety of the interventions

Although it should be noted that the sample size of the study does not allow robust comparisons with fully powered trials (no trends could be reliably observed), the observed safety of the interventions in Santé-AF compares favourably with the usual care pathway. Rate and rhythm control medications are associated with side effects including nausea and vomiting, dizziness, gastro-intestinal disturbance, xerostomia, palpitations, dyspnoea, fatigue and a higher rate of all-cause mortality [18, 53, 18], while minor procedures including electrical cardioversion have been found to cause brady-arrhythmias and increased risk of cardiovascular hospitalisation [53, 18] and a range of periprocedural complications [53]. By comparison, the safety of acupuncture provided during Santé-AF is in accordance with the profile and extent of adverse reactions in recent studies [18, 53]: of 85 treatments given, eight reported bruising, fatigue and post-treatment tiredness that were minor, transient and self-limiting.

Although the safety of isolated nutritional strategies has been investigated to some extent, the safety of an intervention that combines strategies does not appear to have been a topic of investigation; accordingly, there is no research against which to contextualise Santé-AF’s findings.

The adverse reaction of one participant to their nutritional therapy regime divided opinion between the Trial Steering Committee, the participant and the participant’s GP, as to whether this was in fact an adverse reaction to NT or an idiopathic reaction to prescribed medication coupled with unaccustomed exertion. The effects were described by the participant as “inconvenient and detracted from my quality of life quite considerably” (4/142/NT) but were reversible and did not conform to the criteria for a Serious Adverse Event [18]. By these criteria, there would seem to be no substantial evidence of harms associated with the NT intervention and therefore no recommendation to remove NT from a future trial.

### Statistical basis for progression

The sample size for the study was calculated to provide a statistical justification for (non-)progression to a large-scale trial [17], but the non-normal data distribution in Group C meant it was not viable to conduct statistical tests to verify the promise of treatment effects. However, 95.0% of participants in the intervention groups reported perceived positive effects on both AF and a range of other symptoms, and within-group differences in the intervention groups broadly aligned with the difference targeted between the control and each intervention group at follow-up (Table 5).

### Limitations of this study

The study’s unblinded design means there is a risk of bias, which is compounded by the use of self-reported data. In particular, participants’ relationships with their practitioners and the principal investigator may have led to a form of reporting bias in which participants report what they consider to be favourable outcomes, influenced by the relationship in question [53]. It is recommended that a future trial should emphasise to participants the confidentiality of reporting, and both data collectors and outcomes assessors should be blinded to treatment allocation.

The single follow-up for Santé-AF was positioned at the end of treatment, approximately 3 months from the start of intervention; a longer follow-up was not possible due to the delays caused by COVID-19. This represents a significant limitation of this feasibility study, with possible overestimation of retention rates. Trials with too short a follow-up also produce uncertainties regarding whether short-term effects persist and may not detect all adverse responses occurring after the follow-up period. Additionally, there is strong evidence to show that the treatment effects of acupuncture are maintained over durations of up to 12 months [18, 53], and the longitudinal effects of NT have not been investigated to date. Therefore, it is recommended that a future trial should include longer follow-ups, with a more conservative estimate of retention rates than those demonstrated by this study. The effect of including one or more longer follow-ups remains an area of uncertainty for a future trial’s feasibility.

This was a single-centre feasibility study and as such has limited generalisability to a future trial; some parameters, including participant recruitment, require cautious interpretation when applied to multiple alternative sites/local areas. It is recommended that a future trial should be designed as a multi-centre trial.

The scope of nutritional therapy in the study was impacted by funding, which did not allow for supplementation or functional testing, both core elements of NT practice. This would introduce a potential limitation in the real-world generalisability of a future trial’s findings, and it is recommended that supplementation and functional testing should be funded where indicated by a personalised nutrition strategy. For this feasibility study, the effect of adding these two elements remains an area of uncertainty for a future trial.

## Conclusion

This is the first study to investigate the feasibility of a future trial of acupuncture and nutritional therapy for symptoms and HRQoL in atrial fibrillation. It has found that the design of a future large-scale trial of acupuncture and nutritional therapy for symptoms and HRQoL in atrial fibrillation is feasible with regard to participants’ willingness to take part, retention, intervention acceptability, study assessment acceptability and overall acceptability of participation. This was broadly comparable or better than similar feasibility studies in the dimensions of recruitment and retention, and acceptability of study assessments.

Adjustments to eligibility criteria are recommended to enfranchise a study cohort more representative of the general AF population in the UK. Other adjustments are also recommended, including holding study assessments in person to increase measurement validity and acceptability, incorporating longer follow-ups to detect the effect of the interventions over time and introducing a full scope of nutritional therapy practice to increase generalisability of a future trial’s results to real-world settings. To increase feasibility, it is also recommended to remove trial processes relating to COVID-19, unless a future trial takes place under similar pandemic conditions. Remaining uncertainty exists around the effect of reversion to non-COVID-19 practice in interventions and study assessments, and the incorporation of a recommended longer follow-up. There are no current plans to conduct a future large-scale trial.

## Supplementary Information

Supplementary Material 1. Eligibility criteria for participants. Description of data: List of inclusion and exclusion criteria for participants in the Santé-AF study, with justifications.

Supplementary Material 2. Survey of recent comparable trials. Description of data: Results of survey of recent comparable trials to set progression thresholds.

## Data Availability

All materials and data generated and analysed during this study are available from the corresponding author upon reasonable request.

## Abbreviations

A: Acupuncture (when used in participant ID code, i.e., 2/163/A)
AF: Atrial fibrillation
AFEQT: Atrial Fibrillation Effect on Quality of Life questionnaire
BAcC: British Acupuncture Council
BANT: British Association for Nutrition and Lifestyle Medicine
BCE: Before Common Era
BMI: Body mass index
CNHC: Complementary and Natural Healthcare Council
COPD: Chronic obstructive pulmonary disease
CPRD: Clinical Practice Research Datalink (UK database of clinical data)
ECG: Electrocardiograph monitor/reading
GP: General practitioner (i.e., primary care practitioner)
HRA: Health Research Authority (UK)
HRQoL: Health-related quality of life
IQR: Interquartile range
ITT: Intention to treat
mmHg: Millimetres of mercury (blood pressure measurement)
NHS: National Health Service (UK)
NICE: National Institute for Health and Care Excellence (UK)
NIHR: National Institute for Health Research
NT: Nutritional therapy, nutritional therapist
PIC: Participant Identification Centre (primary care practices)
PPE: Personal protective equipment
REC: Research Ethics Committee
RTA: Reflexive thematic analysis
SMS: Short messaging service (i.e., text message via mobile phone)
TCM: Traditional Chinese Medicine
TENS: Transcutaneous electrical nerve stimulation
UC: Usual care (group)

## Declarations

### Registration and protocol

The study was registered on the ISRCTN trials registry on June 04, 2020, registration number 13671984. The trial protocol is available via the Open Science Framework at https://osf.io/kexra.
