# Supplementary material for: Sessions of acupuncture and nutritional therapy evaluation for atrial fibrillation (Santé-AF): a randomised feasibility study
Source: Pilot Feasibility Stud. 2025 Feb 25;11:21. doi: 10.1186/s40814-025-01604-w (PMC11854018; doi:10.1186/s40814-025-01604-w)
Supplement: Supplementary file 1 — Supplementary Material 1. Eligibility criteria for participants. Description of data: List of inclusion and exclusion criteria for participants in the Santé-AF study, with justifications. [file 40814_2025_1604_MOESM1_ESM.docx]

Supplementary file 1

Eligibility criteria for participants

| # | Inclusion criteria (participants) | Justification |
| --- | --- | --- |
| INC1 | Aged ≥ 45 and ≤70 | Age range with greatest prevalence of AF |
| INC2 | Diagnosis of AF ≥ 6 months and ≤ 60 months previously | To coincide with an anticipated ‘settling in’ post-diagnosis period in which conventional treatment has become stable |
| INC3 | Stroke prevention measures offered/applied where indicated | To reduce risk of stroke (National Institute for Health and Care Excellence, 2021) and maintain safety of study participants |
| INC4 | Self-detectable paroxysmal atrial fibrillation of at least weekly frequency | Necessary to reliably complete 7-day self-report diary |
| INC5 | Owner of, and able to use, a mobile phone | For study communication and compliance prompting |
| INC6 | Home broadband of sufficient capacity to sustain NT appointments and study assessments | For nutritional therapy appointments (if allocated to this group) and online study assessments |
| INC7 | Owner of, and able to use, a device capable of video-conferencing OR happy to use study iPad | For online study assessments |
| INC8 | Willing to have acupuncture or nutritional therapy alongside usual care, or usual care alone | To comply with intervention requirements |
| INC9 | Willing to travel to attend appointments for acupuncture *(travel expenses subsidised)* | To attend acupuncture appointments if allocated to that group |
| INC10 | Willing to wear a CardioSTAT® ambulatory ECG monitor for 7 days x 2 times over the course of approximately three months, then return in reply-paid envelope to the manufacturer for data analysis | To provide AF monitoring data |
| INC11 | Speak/understand English well enough to engage meaningfully with interventions and assessments (researcher’s judgement) | No budget to provide translation |
| INC12 | Able to give informed consent | To comply with ethical requirements |

| # | Exclusion criteria (participants) | Justification |
| --- | --- | --- |
| EXC1 | Diagnosed with valvular AF | Type of AF is not amenable to study interventions |
| EXC2 | Pacemaker, implantable defibrillator, neurostimulator, any other type of active implantable device | Contraindicated to CardioSTAT® |
| EXC3 | Diagnosed with kidney disease levels 4 or 5 | Contraindicated to nutritional therapy |
| EXC4 | Diagnosed with terminal or severe illness | To reduce patient burden/reduce loss to follow-up due to illness |
| EXC5 | Diagnosed with any blood clotting disorder | Contraindicated to acupuncture |
| EXC6 | Diagnosed (including self-diagnosis) with any condition or disorder contraindicating suitable moderate exercise | Contraindicated to exercise |
| EXC7 | Diagnosed (including self-diagnosis) with any eating disorder past or present | Contraindicated to nutritional therapy |

*Table continued overleaf*

| # | Exclusion criteria (participants) | Justification |
| --- | --- | --- |
| EXC8 | Pregnant or trying to conceive | For safety of pregnancy |
| EXC9 | Currently taking part in other research rendering participant unable to have either intervention, or which is likely to affect study outcomes, or which renders it unsafe for participant to continue | For safety of participant and integrity of research |
| EXC10 | Currently having, or have had in the last six months, a course of acupuncture or nutritional therapy | Avoidable confounding factor |
| EXC11 | Classified as shielding, clinically vulnerable or clinically extremely vulnerable with regard to COVID-19, or living with or bubbled with anyone in any of these categories (National Health Service, 2022b) | To protect participant and household against increased risk of cross-infection with COVID-19 |
| EXC12 | Regularly using a TENS machine or receiving any kind of energy delivery therapy to the upper torso (diathermy therapy, diagnostic or therapeutic ultrasound, radiation therapy, electro-surgery or x-ray) | Contraindicated to CardioSTAT® |
| EXC13 | Any other clinical reason why patient should be excluded, in the clinical judgement of the patient’s GP | To protect patients |
